# Homœopathic League Tract, No. 19

**Published:** 1888-08

**Authors:** 


Homœopathic League Tracts, No. 19. "The Gains of Medical Liberty in Fifty Years."

The purport of this tract is explained by its title. It tells of the gains for the pas half century. They have been very great. The *Second Annual Report of the Homœopathic League* shows how great an interest is taken in the work. Yet it *should* be far greater, for these tracts are of undoubted value to Homœopathy.

